# Research and Application of Kupffer Cell Thresholds for BSA Nanoparticles

**DOI:** 10.3390/molecules28020880

**Published:** 2023-01-16

**Authors:** Huanhuan Guo, Zongguang Tai, Fang Liu, Jing Tian, Nan Ding, Zhongjian Chen, Shen Gao

**Affiliations:** 1 Department of Pharmacy, Changhai Hospital, Naval Medical University (Second Military Medical University), Shanghai 200433, China; 2 Department of Gastroenterology, Changzheng Hospital, Naval Medical University (Second Military Medical University), Shanghai 200433, China; 3 Shanghai Skin Disease Hospital, School of Medicine, Tongji University, Shanghai 200443, China

**Keywords:** nanoparticles, drug delivery, breast cancer, Kupffer cell, albumin-bound paclitaxel (ABP), bovine serum albumin (BSA)

## Abstract

Over the past decade, the dose of nanoparticles given to solid tumors has remained at a median of 0.7% of the injected dose. Most nanoparticles are trapped in a mononuclear phagocyte system (MPS), of which 85% are Kupffer cells. In our study, threshold doses of bovine serum albumin (BSA) nanoparticles were investigated for the uptake of Kupffer cells in vitro and in vivo. The antitumor effect and safety of albumin-bound paclitaxel (ABP) were improved by using threshold doses of BSA nanoparticles. We found a threshold dose of 20,000 nanoparticles per macrophage uptake in vitro and a saturation dose of 0.3 trillion nanoparticles in tumor-bearing mice. In vivo efficacy and safety evaluations demonstrated that the threshold doses of blank BSA nanoparticles could significantly improve the efficacy and safety of ABP against tumors compared with ABP alone. In this study, the delivery efficiency of ABP was improved by using blank nanoparticles to saturate Kupffer cells, which provided a new approach to studying the Kupffer cell saturation threshold and thus a new scheme for improving the curative effect of ABP.

## 1. Introduction

The unique benefits of nanotechnology for drug delivery, diagnosis, and imaging, as well as the creation of synthetic vaccines and tiny medical devices, represent a large part of the growing interest in using it to treat cancer [1]. Some of these properties of nanotherapies, such as increased circulation and reduced toxicity, are already in use today, while others have shown great potential in clinical trials [2,3]. Therefore, the accurate delivery of nanoparticles to solid tumors is critical for clinical diagnosis and treatment [4,5]. It can not only increase the accuracy of diagnosis but also improve the therapeutic effects of nanomedicines and reduce the off-target effects and side effects of nanoparticles [6]. However, a meta-analysis summarizing studies over the past decade showed that only 0.7% (median) of the injection dose of nanoparticles was successfully delivered to solid tumors [4]. The unique organ structure and blood flow characteristics facilitate the aggregation of nanoparticles in reticuloendothelial system (RES) organs and reduce the delivery efficiency of nanoparticles. The liver is the largest organ of RES, which ingests a large proportion of nanoparticles [7]. At the same time, most nanoparticles are trapped in the mononuclear phagocyte system (MPS) [8,9], of which 85% are Kupffer cells [10,11].

Many researchers have improved targeted delivery efficiency by modifying the physical chemistry properties of nanoparticles, such as particle size [12], surface chemistry [13,14], potential, etc. [5,15]. Another strategy is to increase the delivery efficiency of nanoparticles by reducing RES uptake. For example, strategies for macrophages to reduce the phagocytosis of PEGylated nanoparticles include being killed by chlorophosphonate liposomes [8,16], being inhibited by RES inhibitors [17], and being saturated with high-dose blank nanoparticles [18,19]. The application of RES saturated with blank nanoparticles was proposed as early as 1983 [20]. It has been demonstrated to increase the accumulation of drug-loaded nanoparticles and to improve the therapeutic effect of drug-loaded nanoparticles, but the specific dose and research method of preinjection blank nanoparticles have not been determined. Subsequently, Ouyang et al. proposed a saturation threshold of 100,000 nanoparticles for the uptake of gold nanoparticles by macrophages in vitro, demonstrating the existence of kinetic saturation in the engulfment of nanoparticles by macrophages [21]. The use of nanoparticle concentrations as a unit of dose rather than a mass concentration is a bold reform in the history of nanoparticle delivery research. Therefore, we used the particle concentration of nanoparticles as a unit of concentration to explore the saturation threshold in relation to the Kupffer cell uptake of bovine serum albumin (BSA) nanoparticles.

Compared with conventional Cremophor EL-based paclitaxel, albumin-bound paclitaxel (ABP) has shown more-pronounced efficacy and fewer side effects in clinical practice [2,22]. For example, the strengths of ABP include a short administration time, a reduction in hypersensitivity reactions, a better overall response and better survival, and improvements in life years gained (LYG) and quality-adjusted life years gained (QALYG) [22]. However, ABP, like most nanomedicines, accumulates in the liver after injection. Therefore, we hope to improve the delivery efficiency and therapeutic efficacy of albumin-paclitaxel by utilizing blank nanoparticles to saturate Kupffer cells, which could lower the dose of ABP and reduce side effects such as allergies and neutropenia.

In the natural fluidic environment of a biological system, drug-loaded nanoparticles swiftly adsorb plasma proteins on their surface, forming a “protein corona”, which profoundly and often adversely affects their residence in the systemic circulation in vivo and their interaction with cells in vitro [23]. It has been shown that protein corona can replace nanoparticles’ engineered surfaces and alter their biological interactions [24]. Nanoparticles that form a protein corona are more likely to be taken up by Kupffer cells, reducing drug bioavailability [7]. BSA is a nanocarrier material that can be loaded with paclitaxel. Thanks to the well-defined structure of the protein containing charged amino acids, BSA nanoparticles may facilitate the electrostatic adsorption of negatively or positively charged molecules. BSA nanoparticles without any modification have a higher potential, are more stable, and more easily form a protein corona. BSA nanoparticles promote the formation of the protein corona, which is more conducive to phagocytosis by Kupffer cells, resulting in the saturation of Kupffer cells.

We focused on saturation thresholds for the uptake of Kupffer cells in vivo and in vitro. Moreover, in a 4T1 breast cancer model, we investigated the effects of saturated doses of blank nanoparticles on tumors and mice. ABP is an albumin-stabilized paclitaxel nanoparticle formulation with a particle size of 130 nm [25]. To better mimic ABP in tumor-bearing mice, we designed BSA nanoparticles with a similar size and carrier to those of ABP to saturate Kupffer cells to a greater extent. We found a threshold dose of 20,000 nanoparticles per macrophage in vitro. In tumor-bearing mice, the liver uptake of blank BSA nanoparticles reached saturation at a dose of 0.3 trillion nanoparticles after 30 min of tail vein injection. The preinjection of BSA nanoparticles significantly increased the antitumor efficacy and safety of ABP in a 4T1 breast cancer mouse model. The results of blood biochemical tests and a pathological examination of the major organs showed that BSA blank nanoparticles could not cause functional or tissue damage in tumor-bearing mice. Compared with ABP alone, preinjection blank nanoparticles reduced tissue damage and improved liver and kidney function.

## 2. Results and Discussion

### 2.1. Preparation and Characterization of BSA Nanoparticles

**Figure 1:**
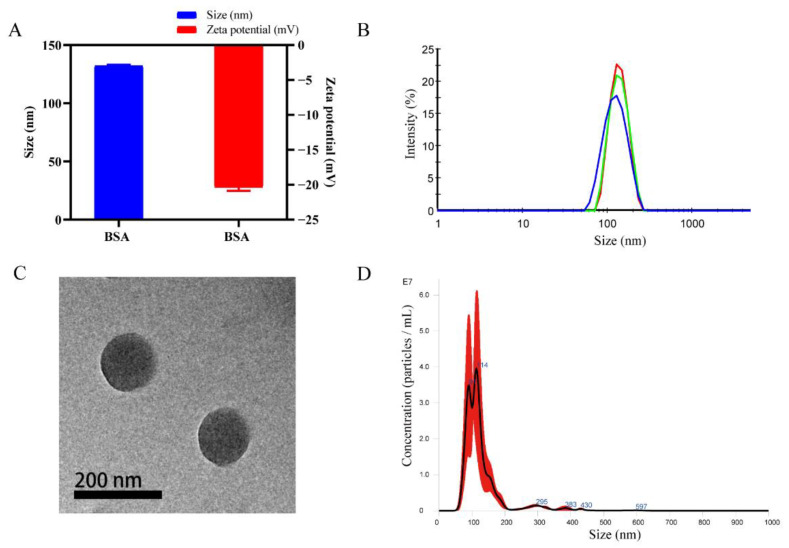
Characterization of BSA nanoparticles. (**A**) Hydrodynamic diameters and zeta potentials of BSA (*n* = 3). (**B**) The hydrodynamic diameter distribution of BSA determined for 3 times. (**C**) Transmission electron microscopy image of BSA. Scale bar = 200 nm. (**D**) The average number of BSA nanoparticles after 1000-fold dilution (*n* = 3).

The particle size and zeta potential of nanoparticles were determined by dynamic light scattering. The data are summarized in Figure 1A. The mean hydrodynamic diameter of BSA was 132.2 ± 0.85 nm. The mean zeta potential of BSA was −20.4 ± 0.5 mV. The mean polydispersity index (PDI) of BSA was 0.118. The hydrodynamic diameter distribution (Figure 1B) showed a peak height of 139.7 nm and a peak width of 34.12 nm. Transmission electron microscopy (TEM) analysis (Figure 1C) showed that the BSA nanoparticles were spherical in morphology, had the same diameter as the hydrodynamic diameter, and were uniformly distributed. It also showed that the nanoparticles were uniform in size and had good stability. After the 1000-fold dilution of BSA nanoparticles, the mean concentration of BSA nanoparticles (Figure 1D) was 2.4 ± 0.25 × 10^9^ particles/mL according to a nanoparticle tracking analysis (NTA). Therefore, the original concentration of the BSA nanosolution was 2.4 ± 0.25 × 10^12^ particles/mL.

### 2.2. Characterization of Threshold In Vitro

#### 2.2.1. Macrophage Uptake Threshold and Safety Assessment In Vitro

In order to investigate the saturation threshold of the uptake of BSA nanoparticles by RAW264.7 cells in vitro, we set up a standard curve in the range of fluorescent nanoparticles and fluorescence intensity (Figure S1) using a multifunctional enzyme marker. The equation of the standard curve is as follows:

Y = 7.347X + 0.3102 (R^2^ = 0.9971) (1)

**Figure 2:**
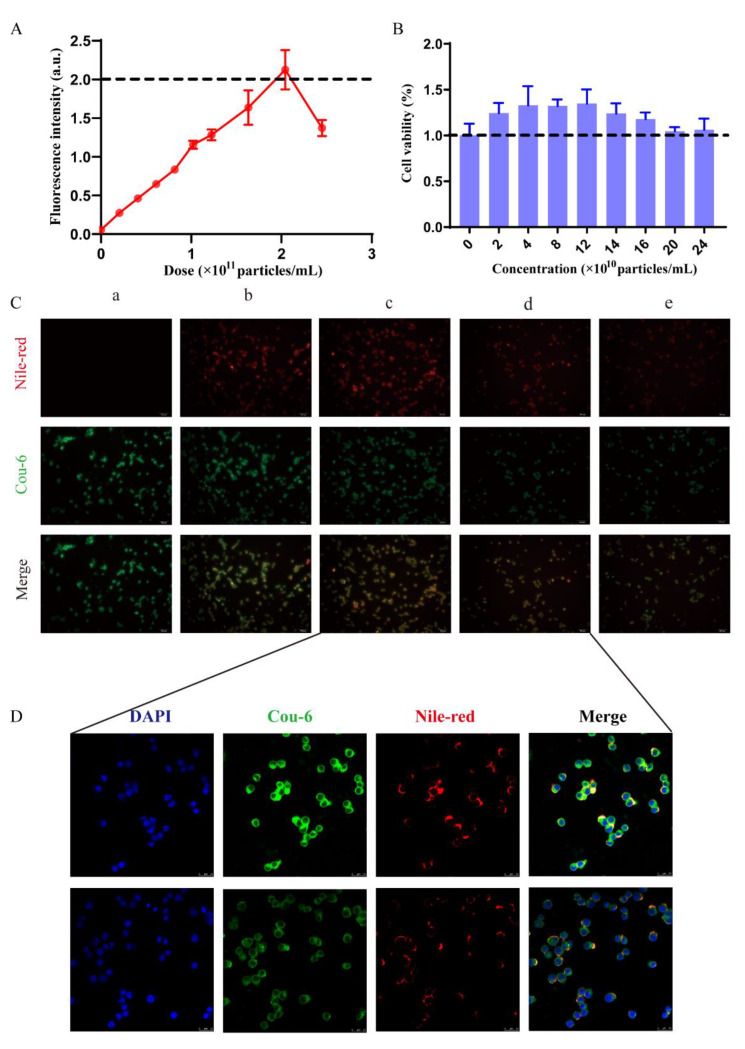
Characterization of threshold in vitro. (**A**) Fluorescence intensity thresholds after 4 h of fluorescent nanoparticle uptake by RAW264.7 cells (*n* = 5). (**B**) CCK-8 assay detects changes in the cell viability of RAW264.7 cells after 4 h of BSA nanoparticle uptake (*n* = 4). (**C**) Representative fluorescence microscopy (FM) images of the saturation of cellular uptake in RAW264.7 cells (*n* = 3). The five concentrations of Nile-red-labeled BSA nanoparticles are a = 0 × 10^11^ particles/mL, b = 1 × 10^11^ particles/mL, c = 2 × 10^11^ particles/mL, d = 2.2 × 10^11^ particles/mL, and e = 2.4 × 10^11^ particles/mL. The concentration of coumarin-6-labeled BSA nanoparticles is 1 × 10^11^ particles/mL. (**D**) Representative confocal laser scanning microscopy (CLSM) images of nanoparticles distribution in RAW264.7 cells during the saturation of cellular uptake.

The fluorescence intensity of RAW264.7 cells after 4 h of fluorescent nanoparticle uptake is shown in Figure 2A. As the dose of fluorescent nanoparticles increased, the fluorescence intensity curve showed an increase followed by a retreat. This result is consistent with the work of Ouyang et al. [21]. We substituted the fluorescence intensity threshold of 2 as the value of Y in the standard curve equation and obtained a threshold of 2.3 × 10^8^ particles for nanoparticle uptake by 10,000 cells. We concluded that the uptake threshold of a macrophage is approximately 20,000 nanoparticles in vitro. According to the study by Ouyang et al., the saturation threshold for macrophage phagocytosis of gold nanoparticles in vitro is 100,000 [21]. This suggests that different nanomaterials and nanoparticle sizes are influential in the saturation dose of Kupffer cells. Therefore, the saturation dose may decrease from the dual effect of the metabolic rate and the uptake rate of Kupffer cells.

The results of the CCK-8 assay are shown in Figure 2B. The cell viability of RAW264.7 cells showed a trend of increasing and then decreasing with increasing BSA concentration. However, the cell viability of RAW264.7 cells was greater than the control group at all doses. According to the fact that macrophages reached saturation after 4 h of uptaking BSA, we observed the changes in cell viability only during this time period. In short, the threshold dose had no toxic or inhibitory effect on RAW264.7 cells but instead had a rather promotive effect.

#### 2.2.2. Characterization of Saturation Phenomena In Vitro

We also observed the uptake saturation in RAW264.7 cells under microscopy. As shown in Figure 2C, the uptake of nanoparticles in the RAW264.7 cell tended to saturate as the concentration of the Nile-red-labeled BSA nanoparticles increased, with a tendency for the red fluorescence intensity to increase and the green fluorescence intensity to decrease. Therefore, combined with the data of Figure 2A, 10,000 cells could be saturated when the dose was 2 × 10^11^ particles/mL and co-incubated for 4 h in vitro. Finally, at doses larger than the saturation threshold, both kinds of fluorescence decreased. To analyze this phenomenon in depth, we used confocal laser scanning microscopy (CLSM) to observe RAW264.7 cells at a threshold dose and an above-threshold dose, respectively. In the CLSM images (Figure 2D), we found that the red fluorescence was distributed in the cytoplasm and cell membrane, whereas the green fluorescence was distributed throughout the cytoplasm. This phenomenon indicates that the uptake of coumarin-6-labeled BSA nanoparticles does not stop, but the uptake rate slows down when the RAW264.7 cell uptake of Nile-red-labeled BSA nanoparticles reaches saturation. Meanwhile, the original Nile-red-labeled BSA was metabolized by RAW264.7 cells after another 4 h. Therefore, red fluorescence and green fluorescence simultaneously decreased.

### 2.3. Characterization of Saturation Phenomena In Vivo

**Figure 3:**
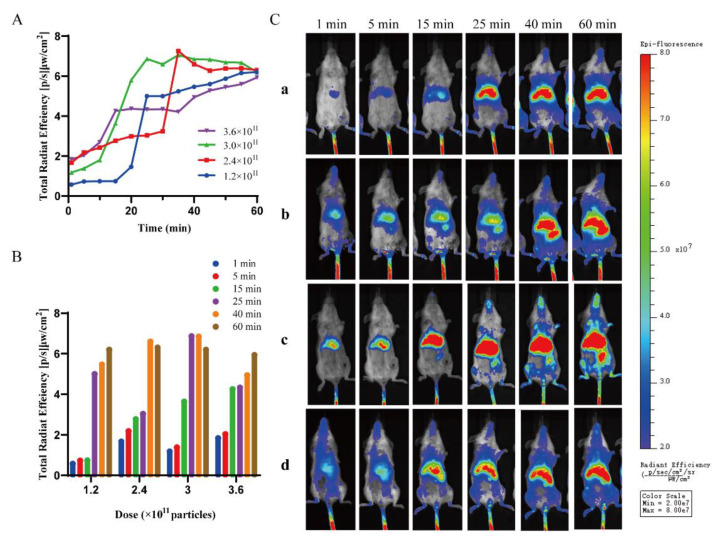
Characterization of threshold in vivo. (**A**) Temporal curve of fluorescence intensity quantification in liver regions. (**B**) Quantitative comparison of fluorescence intensity of liver region at six representative time points at different doses. (**C**) Representative image of live imaging in (**B**). The four concentrations of DiR-BSA nanoparticles are a = 1.2 × 10^11^, b = 2.4 × 10^11^, c = 3 × 10^11^, and d = 3.6 × 10^11^.

Using DiR-labeled BSA nanoparticles, it was observed that the BSA nanoparticles accumulated mainly in the liver region of the tumor-bearing mice. We quantified the fluorescence intensity in the liver region of the mice and used the variation in fluorescence intensity to indicate the extent of nanoparticle uptake in the liver. Although DiR-BSA reaches various organs and tissues with the systemic circulation, we analyzed only the fluorescence intensity for the liver region. Following the quantitative analysis of the fluorescence intensity in the liver region, we plotted the fluorescence intensity versus time (Figure 3A). From this, we found that the fluorescence intensity increases with time, indicating that it takes some time for the liver to take up the nanoparticles. However, after 35 min, the change in the fluorescence intensity level was turned off, indicating that the uptake of nanoparticles by the liver reached a saturation state. For each dose group, the fluorescence intensity was substantially increased for a certain time period.

Subsequently, we compared the relationship between fluorescence intensity and the dose at the same time point (Figure 3B). The fluorescence intensity increased with an increase in dose when the dose was administered within a range of 1.2–3 × 10^11^ particles. When the administration dose reached 3.6 × 10^11^ particles, the fluorescence intensity did not increase correspondingly, indicating that the uptake of nanoparticles by the liver reaches a saturation state at the administration dose of 3 × 10^11^ particles. It has been analyzed that there are approximately 10 million macrophages in mice [26]. The in vitro threshold expanded 10 million times to 0.2 trillion, and the extra 0.1 trillion nanoparticles may circulate in the blood or consumed by macrophages in the lung and spleen.

This saturation phenomenon can also be clearly observed from representative living images (Figure 3C). In addition, the extent to which the nanoparticles tend to accumulate in the peripheral organs was greater as the administered dose increased. When the administered dose was greater than 2.4 × 10^11^ particles, nanoparticles began to accumulate in the spleen. When a dose of 3.6 × 10^11^ particles was administered, nanoparticles began to accumulate in the tumor area at 60 min. Within 1 h after intravenous administration, the DiR red fluorescence was distributed mainly in the liver region, indicating that BSA nanoparticles accumulate in the liver in large quantities. This also suggests that the intravenous dose is the dose that can saturate the liver with BSA nanoparticles.

### 2.4. Application of Saturated Doses of BSA Nanoparticles in Antitumor Efficacy

**Figure 4:**
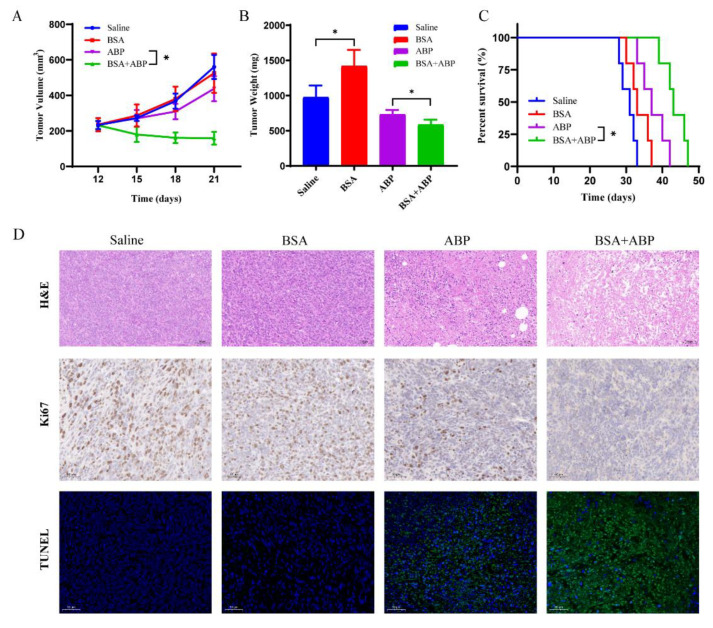
Application of saturated dose of BSA nanoparticles in antitumor efficacy. (**A**) Tumor volumes, (**B**) tumor weights, and (**C**) survival curves of mice following the various treatments. (**D**) Hematoxylin and eosin (H&E), Ki67, and TUNEL staining of tumor tissues from each group. * *p* < 0.05 between groups.

Furthermore, 12 days after the tumor was seeded, the mice were randomly divided into saline, BSA, ABP, and BSA + ABP groups. We evaluated the antitumor efficiency according to the tumor growth volume (Figure 4A), tumor mass (Figure 4B), and survival curve (Figure 4C) of the mice. As shown in Figure 4A, on day 15, the tumor volume of the mice in the BSA + ABP group significantly decreased, and the tumor growth trend was clearly opposite to that of the other control groups. Mice in the ABP group showed a slight decrease in tumor growth volume compared with those in the control group. According to Figure 4B, there was a significant difference in tumor mass between the normal saline group and the BSA group, indicating that BSA acts as a nutrient to promote tumor growth. There was a significant difference in the tumor mass between the ABP group and the BSA + ABP group, suggesting that BSA-saturated Kupffer cells and ABP are synergistic. The median survival of the mice in the BSA + ABP group was extended to 43 days compared with the other groups (Figure 4C). Survival was prolonged in the BSA group compared with the normal saline group, but the difference was not statistically significant. In conclusion, the antitumor effect of ABP was significantly enhanced under the saturation of Kupffer cells by BSA nanoparticles. BSA as a nutrient can promote the growth of tumors, but it does not affect the survival time of the tumor-bearing mice.

To further investigate the antitumor effects of BSA + ABP, histological evaluations and TUNEL assays were performed to determine apoptotic levels within tumor tissue. Furthermore, Ki67 immunohistochemical staining was performed to analyze the proliferation level in the tumor tissues. As shown in Figure 4D, in the tumor tissues of the BSA + ABP group, we observed larger contractile and apoptotic regions with lower proliferation levels than in the other groups. This suggests that the antitumor effect of ABP increases after the BSA nanoparticles have been saturated with Kupffer cells.

### 2.5. Evaluation of the Safety of BSA Saturation Doses in Antitumor Efficacy

**Figure 5:**
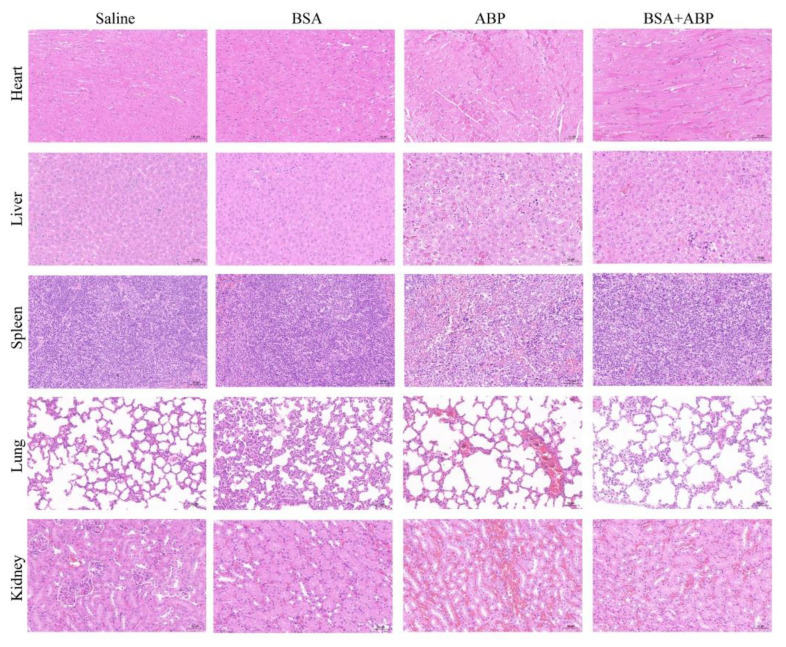
Evaluation of the safety of BSA saturation dose in antitumor efficacy. Histological assessment of the heart, liver, lung, spleen, and kidney.

According to a histopathological analysis (Figure 5), there was no significant tissue damage in either the normal saline group or the BSA group. In the ABP group, there was significant tissue damage to each major organ, including increased interstitial space and interstitial hyperemia. The BSA + ABP group showed less tissue damage compared with the ABP group. As shown in Table 1, serum indices of aspartate aminotransferase (AST) and blood urea nitrogen (BUN) were abnormal in the ABP group, indicating impaired liver and kidney function. At the same time, serum indices in the BSA + ABP and other groups were within the normal range, indicating normal liver and kidney function. There was no significant difference in the body weight change in the mice among the groups (Supplementary Materials: Figure S2). In conclusion, the saturating dose of BSA has good safety and tolerability. BSA-saturated Kupffer cells play a protective role in the antitumor effect of ABP, which significantly improves the safety of ABP.

## 3. Materials and Methods

### 3.1. Materials and Reagents

BSA and DAPI Fluoromount-G were purchased from Yeasen Biotechnology Co., Ltd. (Shanghai, China). In addition, 1,1′-Dioctadecyl-3,3,3′,3′-tetramethylindotricarbocyanine iodide (DiR) and ethanol were purchased from Aladdin Biochemical Technology Co., Ltd. (Shanghai, China). Nile red and coumarin-6 were purchased from Yuanye Biotechnology Co., Ltd. (Shanghai, China). Roswell Park Memorial Institute 1640 medium (RPMI 1640), Dulbecco’s modified Eagle’s medium (DMEM), trypsin, fetal bovine serum (FBS), and 1% penicillin–streptomycin were purchased from Gibco. ABP was purchased from Jiangsu Hengrui Pharmaceuticals Co., Ltd. (Lianyungang, China).

### 3.2. Cell Lines and Animals

RAW264.7 macrophage cells and 4T1 breast cancer cells were purchased from the Institute of Biochemistry and Cell Biology (Shanghai, China). The 4T1 cells were cultured in RPMI 1640 medium, supplemented with 10% FBS and 1% penicillin–streptomycin (10,000 U/mL). RAW264.7 macrophage cells were cultured in DMEM media supplemented with 10% FBS and 1% penicillin–streptomycin (10,000 U/mL). The maintenance medium for RAW264.7 macrophage cells was the DMEM medium, supplemented with 5% FBS and 1% penicillin–streptomycin (10,000 U/mL). All cells were grown in an atmosphere containing 5% CO_2_ and maintained at 37 °C in a humidified chamber (Thermo Fisher Scientific Inc., Waltham, MA, USA). Female BALB/C mice (both 6 weeks old) were purchased from the Animal Care Center of the Second Military Medical University (Shanghai, China) and raised under specific pathogen-free conditions. All experiments were carried out under the guidelines of the Research Center for Shanghai Skin Disease Hospital.

### 3.3. Preparation of the BSA Nanoparticles

BSA nanoparticles were prepared using the ethanol dehydration heat-curing method. We dissolved 25 mg of BSA in 5 mL of water and then slowly added 25 mL of ethanol into the BSA solution. After stirring for 15 min, the mixture was placed in a water bath at 78 °C; and 1 mL of the remaining liquid was heated until it evaporated. We added 29 mL of pure water again, centrifuged it at 13,000 rpm for 1 h, and discarded 29 mL of the supernatant. The above steps were then repeated twice. Finally, 25 mg/mL of BSA-concentrated nanoparticles was obtained. In this process, 0.3 mg of fluorescent dye was added into ethanol to obtain the fluorescent-labeled BSA nanoparticles. BSA was separately marked with Nile red, coumarin-6, and DiR in the follow-up experiments.

### 3.4. Characterization of the BSA Nanoparticles

The hydrodynamic diameters and zeta potentials of the PLGA and BSA were measured using ZetaSizer Nano ZS90 (Malvern Instruments Ltd., Malvern, UK). The morphology of nanoparticles was observed using a Tecnai G2 S-Twin transmission electron microscope (FEI Company, Hillsboro, OR, USA). The number of BSA nanoparticles was determined through the nanoparticle tracking analysis (NTA) technique of NanoSight NS300 (Malvern Panalytical Ltd., Malvern, UK).

### 3.5. Macrophage Uptake Threshold and Safety Assessment In Vitro

Nile-red-labeled BSA nanoparticles were gradient-diluted to 2.4 × 10^10^ particles/mL, 1.92 × 10^10^ particles/mL, 1.44 × 10^10^ particles/mL, 0.96 × 10^10^ particles/mL, and 0.48 × 10^10^ particles/mL by acetonitrile. Then, 100 µL of each gradient concentration was added to a 96-well plate, and 5 replicate wells were set up. Finally, 100 µL of acetonitrile was set as the blank control group. The fluorescence intensity was measured at an excitation wavelength of 530 nm and an emission wavelength of 63 nm by multifunctional enzyme marker (Thermo Fisher Scientific Inc., Waltham, MA, USA). The horizontal coordinate of the standard curve was the gradient concentration, and the vertical coordinate was the fluorescence intensity.

Second, we seeded RAW264.7 cells in 96-well plates (10,000 cells per well) with the DMEM maintenance medium. When RAW264.7 cells were attached (about 4 h), the medium was discarded. Next, 100 µL of Nile red BSA was added to each well after gradient dilution with the maintenance medium. RAW264.7 cells were incubated with the drug-containing medium for 4 h, and then the drug-containing medium was discarded and washed with PBS 3 times. Finally, RAW264.7 cells were lysed with 100 µL of triton × 1. A control group of only 100 µL triton × 1 was then set up. Finally, the fluorescence intensity of each well was measured with a multifunctional microplate reader at an excitation wavelength of 530 nm and an emission wavelength of 635 nm.

In the above process, RAW264.7 cells were co-incubated with blank BSA instead of Nile-red-labeled BSA for 4 h. Afterward, the drug-containing medium was discarded and washed with PBS 3 times. We used the CCK-8 method to detect the cell viability of RAW264.7 cells.

### 3.6. Characterization of Saturation Phenomena In Vitro

We seeded RAW264.7 cells in 96-well plates with 10,000 cells per well. RAW cells were co-incubated with different concentrations of Nile-red-labeled BSA for 4 h, and the medium containing Nile red BSA was discarded and washed 3 times with PBS. Cou-marin-6-labeled BSA at the same concentration was incubated for another 4 h. Finally, the culture medium was discarded and washed 3 times with PBS. Finally, the cellular uptake was observed with a fluorescence microscope. Subsequently, we observed the distribution of fluorescence in RAW264.7 cells by CLSM at a concentration of 2 × 10^11^ particles/mL and 2.2 × 10^11^ particles/mL.

### 3.7. Tumor Inoculation

For tumor inoculation, 7-week-old female BALB/c mice were selected. A syngeneic 4T1 tumor model was established by injecting 1 million 4T1 cells into the right fourth mammary fat pad (recorded as day 0).

### 3.8. Characterization of Saturation Phenomena In Vivo

To investigate the saturated dose of BSA nanoparticles in the liver of mice, we used DiR to trace BSA nanoparticles. After different doses of DiR-BSA were injected into the tail vein of the mice, the mice were photographed every 5 min for 1 h by using a living imaging system (IVIS Lumina II, Caliper life Sciences, Hopkinton, MA, USA). We performed a quantitative analysis of the fluorescence intensity in the liver region of mice. The relationship between fluorescence intensity and time was analyzed, and the saturation time of liver uptake was obtained. The relationship between fluorescence intensity and doses was analyzed, and the saturated dose of liver uptake was obtained.

### 3.9. Application of Saturated Dose of BSA Nanoparticles in Antitumor Efficacy

Tumor-bearing mice were randomly divided into 4 groups (*n* = 10). On day 12, different agents were administered via intravenous tail every 3 days for a total of 3 injections. Each mouse was administered saline at a dose of 100 µL, BSA at a dose of 0.3 trillion, and ABP at a dose of 20 mg/kg. Then, 30 min after the BSA injection in the BSA + ABP group, the ABP injection followed. Tumor length (L) and width (W) were measured every 3 days, starting on day 12. Tumor volume (mm^3^) = L × W^2^/2. On day 21, 5 mice in each group were executed, and their tumor tissue and major organs were excised. The other 5 mice were used to examine the survival period. Efficacy was assessed according to tumor volume, tumor mass, and survival curve in each group.

### 3.10. Evaluation of the Safety of BSA Saturation Dose in Antitumor Efficacy

The safety assessment included body weight monitoring, the H&E staining of major organs, and the assessment of liver and kidney functions. The body weight of the mice was monitored every 3 days starting from day 12. The major organs were immobilized and stained with H&E for histopathological analysis. In addition, serum was collected to determine the levels of alanine aminotransferase, aspartate aminotransferase, albumin, blood urea nitrogen, and creatinine as indicators of kidney and liver function.

## 4. Conclusions

In our study, fluorescently labeled BSA nanoparticles were used to analyze the uptake threshold of nanoparticles by Kupffer cells in vitro and in vivo. Each macrophage uptakes roughly 20,000 nanoparticles in vitro to reach saturation. The dose of BSA administered through the tail vein of mice reached 0.3 trillion nanoparticles, and uptake can reach a saturated state in the liver. These two saturation thresholds were scientifically reasonable. In conclusion, we designed a new method for Kupffer cell uptake saturation, obtained the saturation dose of BSA nanoparticles (130 nm) by following uptake with Kupffer cells, proved that the saturated dose did not damage the main organs and functions of mice, provided an accurate solution for improving the efficiency of delivery, and improved the anticancer effect and safety of ABP.

## Figures and Tables

**Table 1:** Detection results of liver and kidney indexes in serum (*n* = 3).

Group	ALT (U/L)	AST (U/L)	ALB (g/L)	BUN (mg/dL)	CREA (umol/L)
Saline	45.32 ± 3.07	175.01 ± 27.1	32.72 ± 1.44	27.31 ± 1.53	48.71 ± 8.35
BSA	41.87 ± 8.53	178.24 ± 31.50	31.03 ± 2.05	18.24 ± 1.72	49.52 ± 5.58
ABP	36.64 ± 9.72	283.11 ± 122.60	30.48 ± 2.24	40.24 ± 11.89	34.56 ± 9.40
BSA + ABP	35.72 ± 7.00	186.91 ± 21.50	34.13 ± 1.64	23.71 ± 1.24	29.20 ± 5.94
Normal	10.06–96.47	36.31–235.48	21.22–39.15	10.81–34.74	10.91–85.09

Abbreviations: ALT: alanine aminotransferase; AST: aspartate aminotransferase; ALB: albumin; BUN: blood urea nitrogen; CREA: creatinine.

## Data Availability

Not applicable.

## Supplementary Materials

The following supporting information can be downloaded at https://www.mdpi.com/article/10.3390/molecules28020880/s1. Figure S1: A standard curve corresponding to the fluorescence intensity and multiple concentrations of Nile-red-labeled BSA nanoparticles (*n* = 5). Figure S2: Comparison of relative body weights among treatment groups (*n* = 5). ns, *p* > 0.05.
