# Supplementary material for: Research and Application of Kupffer Cell Thresholds for BSA Nanoparticles
Source: Molecules. 2023 Jan 16;28(2):880. doi: 10.3390/molecules28020880 (PMC9864197; doi:10.3390/molecules28020880)
Supplement: Supplementary file 1 [file molecules-28-00880-s001.zip › molecules-2108813-supplementary.pdf]

Research and application of Kupffer cell threshold for BSA nanoparticles

Huanhuan Guo [1†], Zongguang Tai [1†], Fang Liu[2†], Jing Tian [1], Nan Ding [1], Zhongjian Chen [3, *], and Shen Gao [1, *]

1 Department of Pharmacy, Changhai Hospital, Naval Medical University (Second Military Medical University), Shanghai 200433, China
2 Department of Gastroenterology, Changzheng Hospital, Naval Medical University (Second Military Medical University), Shanghai 200433, China
3 Shanghai Skin Disease Hospital, School of Medicine, Tongji University, Shanghai 200443, China
* Correspondence: 1905059@tongji.edu.cn or aajian818@163.com (Z.C.); liu136256@smmu.edu.cn or liullk@126.com (S.G.); Tel.: +86-021-36803021 (Z.C.); +86-021-31162297 (S.G.)
† These authors contributed equally to this work.

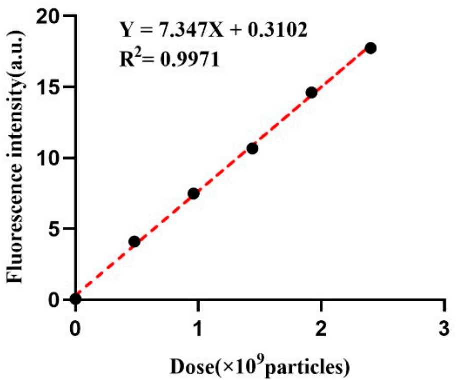

Figure S1. A standard curve corresponding to the fluorescence intensity and multiple concentrations of Nile red-labeled BSA nanoparticles (n=5).

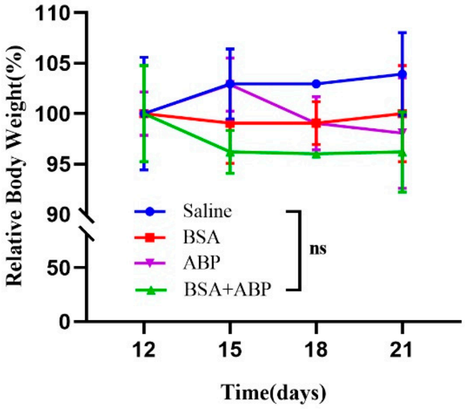

Figure S2. Comparison of relative body weight among treatment groups (n=5). ns, $p > 0.05$.
